# An Analysis of the Relationship between the Modified Theory of Planned Behavior and Leisure Rumination of Korean Employees

**DOI:** 10.3390/ijerph18010320

**Published:** 2021-01-04

**Authors:** Young-Jae Kim, Seung-Woo Kang

**Affiliations:** Department of Physical Education, Chung-Ang University, Seoul 06974, Korea; yjkim@cau.ac.kr

**Keywords:** modified theory of planned behavior, leisure rumination, leisure activity, recovery, work stress

## Abstract

An increasing trend among employees has been to engage in leisure activities, which has been proven to be an effective method of overcoming work stress. As a result, employees are doing “Other Things” (i.e., non-work activities) as a way to relieve stress. Based on the existing studies on rumination, this study considered doing “Other Things” as a new concept of “leisure rumination” and identified its influence as a means to help employees recover from work stress. Accordingly, this study provided basic data on the meaning of leisure activities and leisure rumination in office workers who suffer from failure to recover from work stress using partial least squares structural equation modeling. This study was conducted on employees residing in the Seoul metropolitan area and the Gyeongsang-do district in November 2019 through a structured questionnaire. The results of this study verified the significance of leisure rumination and the possibility of it being utilized as a practical research tool for leisure activities. Our findings may be considered when planning interventions for work addiction and burnout through leisure rumination.

## 1. Introduction

Recently, “recovery” has been recognized as an important factor in the field of occupational health psychology for employees. Recovery from work refers to the process by which people reduce their stress levels and renew the resources and energy lost during work situations [1,2,3]. Recovery was reported to be a regulating factor that prevents work addiction and job stress from leading to negative consequences [4,5,6,7]. For example, employees in Korea take time off from their busy schedules to engage in leisure activities and pursue a balance in work and life in various ways, in an effort to make the most of their time [8].

Leisure activity is known to have a great influence on physical and mental health [9,10,11]. As such, leisure activity is a catalyst for social development and living a healthy life, and positively affects the lives of modern people in areas such as interpersonal relationships, community spirit, life satisfaction, stress, and self-realization [12,13,14,15]. In other words, Korean employees engage in leisure activities as a way to relieve their work stress. It has been found that office workers recover energy by lowering their levels of physical and mental stress, which is a recovery process, by getting away from their work responsibilities, enjoying their time, and engaging in leisure activities [16].

Employees also tend to utilize “Other Things” as an easy way to recover from stress [17]. The term “Other Things” refers to actions that are completely irrelevant to one’s job [18]. With this concept, Young-Jae Kim (2019) formulated a new concept of leisure rumination that occurs in everyday life, based on existing studies on rumination. Considering “leisure rumination” as a phenomenon of doing Other Things, Kim’s study was conducted based on the question of whether it is a work-related phenomenon occurring during leisure activities or in daily life while seeing and thinking of interesting things. Previous research found that Other Things usually done during work hours included “Internet searches,” “instant messaging,” “Internet shopping,” “teatime,” “smoking,” “social network service activity,” “sleeping,” and “playing games” [19,20]. Studies conducted by Kang and Kim [21] and Wendsche and Lohmann-Haislah [22] showed that modern people are able to mentally recover from work stress through Other Things, namely, non-working hours, and that thoughts of leisure activities help solve actual psychological and emotional problems. Employees were found to perceive doing Other Things as having a positive effect on conducting work tasks, stating that they “improve work efficiency by providing moderate rest” and “promote close relationships with colleagues” [23,24].

Even though employees tend to engage in leisure activities as a form of recovery [25], there is currently a lack of research related to leisure rumination. Kim [17] indicated the possibility of extending their research to the relationship between rumination and the factors of Ajzen’s [26,27] theory of planned behavior—the results of which could be applied to interventions targeting behavioral changes for the physical and psychological health of people suffering from ruminating thoughts. In addition, according to Kim’s study [28], achievement and cause are valued in Korean personalities and social activities because face-saving is dictated by social rules and behavioral patterns and is characterized by the consciousness of others, formality, and shame [29,30]. Kim argued that when engaged in face-saving, these characteristics are influencing factors created by uncertainty avoidance, and that face-saving is a very important psychological variable in Korea [31,32]. Therefore, in relation to the general characteristics of Koreans and the leisure activities they enjoy, face-saving is a factor controlled by social rules and behavioral styles, and achievements are emphasized. Koreans tend to be conscious of others and respond sensitively to formal consciousness and embarrassment. Therefore, face-saving is considered an important aspect of leisure behavior in Korea because of evaluation by others, and we sought to confirm whether it is a factor of engaging in leisure behavior.

Therefore, this study aimed to provide basic data on employees’ leisure rumination by identifying the factors related to engaging in leisure rumination about things that Koreans think about in their daily lives and the significance of the factors of the modified theory of planned behavior, by using partial least squares (PLS) structural equation model analysis.

## 2. Research Model and Hypotheses

**Figure 1:**
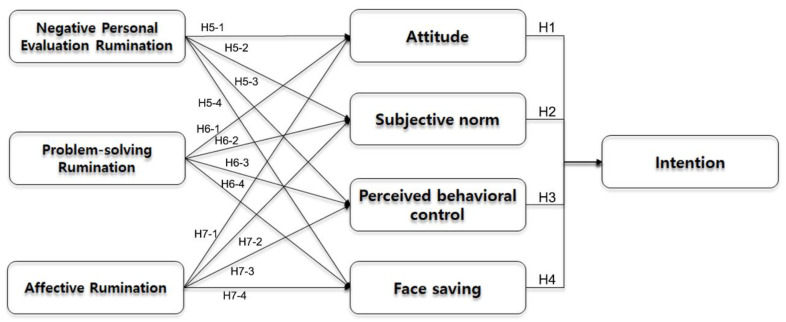
Research model.

The objectives of this study were to analyze the effects of the four cognitive factors associated with leisure rumination among Korean employees (attitude toward leisure rumination, subjective norm, behavioral control, and face-saving) on leisure activity intention based on Ajzen’s [27] theory of planned behavior and Kim’s [33] modified theory of planned behavior and to determine the relationship between individuals’ general disposition toward leisure activities and the four influencing factors. A diagram of the research model is shown in Figure 1. To verify the effects of leisure rumination on leisure activity, leisure rumination of employees (hypotheses H1 to H7-4) was included as the antecedent variable of the modified theory of planned behavior.

### 2.1. Modified Theory of Planned Behavior

This study utilized Young-Jae Kim’s [33] modified theory of planned behavior and Ajzen’s [27] theory of planned behavior. Intention was verified as a sustaining factor for individuals’ leisure activities. Studies on the intention of an action are significant because, usually, the stronger the intention, the higher the prospect of performing an action [27]. Second, attitude refers to the degree of favorable or undesirable assessment of individuals continuing their leisure activities. In other words, attitude was an antecedent factor of intention in this study. As such, attitude seems to have a positive effect on continuous use intention, stronger than that of subjective norm. Leisure activities were indicated as a generally good source of happiness, satisfaction, pride, and achievement in a moderate level of satisfaction in life [34]. Therefore, this study aimed to identify the influence of intention on employees’ attitudes toward leisure activities. Third, subjective norm refers to an individual’s belief in whether or not people or groups significant to this person approve and support a particular leisure activity. Previous studies showed that subjective norm is a positive antecedent factor of intention [35,36]. Thus, this study identified the effects of leisure rumination on subjective norms. Finally, behavioral control refers to the degree of difficulty an individual recognizes in relation to leisure activities. In other words, behavioral control is an important predictor of intention, and studies conducted by Gu et al. [37] and Galla and Duckworth [38] confirmed the effect of behavioral control on the successful achievement of goals. Hence, this study identified the influence of leisure rumination on the perception of control for the leisure activities of employees.

**Hypothesis 1** **(H1).** *Attitude toward leisure activities will have a positive effect on leisure activity intention.*

**Hypothesis 2** **(H2).** *Subjective norm for leisure activities will have a negative effect on leisure activity intention.*

**Hypothesis 3** **(H3).** *Perceived behavioral control of leisure activities will have a positive effect on leisure activity intention.*

#### Face-Saving

Face-saving is a newly verified factor in Young-Jae Kim’s [33] modified theory of planned behavior and refers to being conscious of others about one’s perception, thereby affecting the trust of interpersonal relationships in general. In Western cultures, face-saving is about managing one’s self-image and is self-centered in nature [39,40,41]. In Eastern cultures, however, face-saving is described as showing or protecting one’s image depending on one’s relationships with others as well as the situation [42,43,44,45]. Korean employees are characterized by modest behavior, especially the avoidance of negative reinforcement and pursuit of attention. Therefore, this study identified the influence of face-saving on leisure rumination for employees.

**Hypothesis 4** **(H4).** *Face-saving for leisure activities will have a negative effect on leisure activity intention.*

### 2.2. Leisure Rumination

Studies have examined the effect of the regulation of psychological factors, such as depression, anxiety, and psychological well-being, on rumination [46,47]. Although rumination simply means repetitive thinking and essentially “chewing the cud“ in a cognitive sense [48], the criteria for the term are ambiguous. In this study, the criteria for the Korean version of leisure rumination [17] were set as described below.

#### 2.2.1. Negative Personal Evaluation Rumination

Negative personal evaluation rumination refers to a potential threat of receiving negative recognition and evaluations of one’s leisure activities from others in excessive work conditions [17,49]. A previous study found that by focusing more on their own thoughts and feelings than the judgments of others, individuals experienced fewer negative emotions, which resulted in lower stress levels [50,51]. In other words, we experience negative memory biases that happen daily [52]; it can be said that the problem can be solved in the short term by considering pleasure through leisure activities. That is, those who experience negative rumination have negative autobiographical memories [53], more easily connect to negative self-related information [54], and may have a hard time forgetting negative events [55,56,57]. Such problems can be solved through relaxation via leisure activities. Better recollections can be predicted through participation in the reflection of naturally occurring negative life events [49,58]. Therefore, negative personal evaluation rumination is expected to have a positive effect on one’s continuation of leisure activities through the judgment of others at work or in daily life.

**Hypothesis 5-1** **(H5-1).** *Negative personal evaluation rumination on leisure activities will have a positive effect on attitude.*

**Hypothesis 5-2** **(H5-2).** *Negative personal evaluation rumination on leisure activities will have a positive effect on subjective norm.*

**Hypothesis 5-3** **(H5-3).** *Negative personal evaluation rumination on leisure activities will have a positive effect on perceived behavioral control.*

**Hypothesis 5-4** **(H5-4).** *Negative personal evaluation rumination on leisure activities will have a positive effect on face-saving.*

#### 2.2.2. Problem-Solving Rumination

Problem-solving rumination refers to employees experiencing psychological relaxation through thoughts of leisure activities while working to solve work-related problems [59]. Many previous studies have shown a significant correlation between rational problem-solving rumination and depression. First, it was shown that rational problem-solving ability is an important predictor of positive problem orientation [60], which is distinguished from the ability to implement problem-solving strategies in real life [61,62]. In addition, cognitive and behavioral reactions to avoid negative environments and negative personal life events, and active problem-solving and social problem-solving abilities, have been shown to influence all areas of life, including interpersonal and workplace relationships [63,64,65]. In other words, thinking about work can have a positive effect on “innovation” and “creativity” [66,67]. Based on a meta-analysis of research on mood and creativity, Baas et al. [66] suggested that activating a mood state with a positive focus (i.e., happiness) can lead to increased creativity. Therefore, high scores on measures of problem-solving rumination are expected to have a positive impact on participants’ antecedents to leisure activity.

**Hypothesis 6-1** **(H6-1).** *Problem-solving rumination on leisure activities will have a positive effect on attitude.*

**Hypothesis 6-2** **(H6-2).** *Problem-solving rumination on leisure activities will have a positive effect on subjective norm.*

**Hypothesis 6-3** **(H6-3).** *Problem-solving rumination on leisure activities will have a positive effect on perceived behavioral control.*

**Hypothesis 6-4** **(H6-4).** *Problem-solving rumination on leisure activities will have a positive effect on face-saving.*

#### 2.2.3. Affective Rumination

Affective rumination refers to perceptions that makes one feel positive emotions through thoughts of leisure activities during work or in daily life [18,68,69]. Previous studies [70,71,72] suggested that affective rumination has a momentary effect on the level of positive and negative influences in one’s daily life. This implies that while stress, depression, and anxiety are caused by rumination immediately after negative events occur, positive rumination helps to reduce these negative effects. After experiencing such a complicated or stressful experience, it was shown that some people solve work-related problems by thinking about leisure activities [73]. However, previous research suggested that people can actually make their thoughts more accessible by attempting to push them out of their consciousness [74,75]. Thus, this study aimed to demonstrate that affective rumination on leisure activities will have a positive effect on aspects of one’s daily life, including stress management, sleep, and critical thinking skills.

**Hypothesis 7-1** **(H7-1).** *Affective rumination on leisure activities will have a positive effect on attitude.*

**Hypothesis 7-2** **(H7-2).** *Affective rumination on leisure activities will have a positive effect on subjective norm.*

**Hypothesis 7-3** **(H7-3).** *Affective rumination on leisure activities will have a positive effect on perceived behavioral control.*

**Hypothesis 7-4** **(H7-4).** *Affective rumination on leisure activities will have a positive effect on face-saving.*

## 3. Materials and Methods

### 3.1. Sample and Participants

The participants of this study were employees residing in the Seoul metropolitan area and Gyeongsang-do district, from 1–15 November 2019. To find out the relationship between the modified plan behavioral theory and leisure rumination, a paper-and-pencil questionnaire was utilized, based on a structured questionnaire. After explaining the purpose of the questionnaire in writing, written informed consent was obtained from each participant. The questionnaire was then distributed to a total of 400 participants using convenience sampling. A total of 380 copies of the questionnaire were collected and 358 copies (male = 190, female = 168) were used in this study after excluding 22 unresponsive and undependable copies. The average age of participants was 35.92 years (SD = 9.74), and the types of leisure activities participants engaged in were cultural art (23.7%), sports (33.5%), tourism activities (10.9%), recreational activities (8.1%), relaxing activities (20.9%), and social and other activities (2.8%). For household income satisfaction, 12 participants responded “very satisfied” (3.4%), 108 participants responded “satisfied” (30.2%), 200 participants responded “normal” (55.9%), 37 responded “dissatisfied” (10.3%), and 1 participant responded “very dissatisfied” (0.3%).

During the study, special attention was given to avoiding potential physical or mental harm to the participants. In general, research participants may suffer psychological harm in the process of social research; thus, the researchers in the present study were careful and vigilant regarding even the smallest risk [76]. In particular, the questionnaire was constructed so that the study participants would not experience uncomfortable or unpleasant feelings, and the concept of informed consent was formulated to establish an ethical norm of voluntary participation in the study and a lack of harm to participants [77]. This code encouraged voluntary participation with a full understanding of the possible risks of participating in research studies. Additionally, to conduct such research, we engaged in discussions with two experts in the field of leisure and social psychology and three doctors when constructing and verifying the content validity of the questionnaire. Finally, the anonymity and confidentiality of the participants in this study were ensured.

### 3.2. Data Analysis

According to Howell and Higgins [78], PLS was appropriately used in the stage of theory development through an explanation of the feasibility of the whole model and cause and effect. This study used a PLS structural equation software program to determine the major variables that explain and predict the leisure rumination behavior of participants who engage in leisure activities. Therefore, the PLS structural equation model was applied as the data analysis method in this study. The collected data were analyzed with Smart PLS 3.0 M3 (SmartPLS GmbH, Bönningstedt, Germany) and SPSS for Windows (Version 25.0, IBM, Armonk, NY, USA), using the PLS structured software.

### 3.3. Measurements

Based on the leisure rumination scale of Kim [17], factors used by Kim [33] and Ajzen [27] in the modified theory of planned behavior were modified to fit the purpose of this study and used as the research tool. The modified theory of planned behavior used in this study adds to the theory’s other factors the Korean element of face-saving, as validated by Kim [33]. Details for each factor are described below.

#### 3.3.1. Leisure Rumination Scale

In this study, the identifying factors that confirm thoughts of leisure activities in the context of the Korean working population were organized into questions through the leisure rumination scale [17]. A total of 12 questions related to positive emotion (four questions), negative personal evaluation (four questions), and problem-solving rumination (four questions) were used. The results of a confirmatory factor analysis on leisure rumination are provided in Table 1. In addition, the Heterotrait–Monotrait ratio (HTMT) values were all less than 0.90; thus, it was judged that HTMT.90 secured discriminant validity between all latent variables [79]. The composite reliability values of problem-solving rumination, negative personal evaluation rumination, and affective rumination that explain leisure rumination were all found to have internal consistency reliability, without problems of discriminant validity, since they met the Fornell–Larcker criterion shown in Table 2 [79].

#### 3.3.2. Modified Theory of Planned Behavior

To measure the modified theory of planned behavior by leisure type, the factors used in Kim’s study [33], based on Ajzen’s [27] study, were modified to fit the purpose of the present research. The questionnaire used a 5-point Likert scale consisting of a total of 16 questions regarding five factors: attitude (four questions), intention (three questions), face-saving (three questions), subjective norm (three questions), and behavioral control (three questions). The results of the confirmatory factor analysis for modified planned behavior are shown in Table 3. The composite reliability values of intention, attitude, subjective norm, behavioral control, and face-saving that explain modified planned behavior were all found to have internal consistency reliability, as seen in Table 3, and without problems of discriminant validity, since they met the Fornell–Larcker criterion shown in Table 4 [79]. In addition, the HTMT values were all less than 0.90; hence, it was judged that HTMT.90 secured discriminant validity between all latent variables [79].

## 4. Results

### Results of PLS Structural Equation Analysis

**Figure 2:**
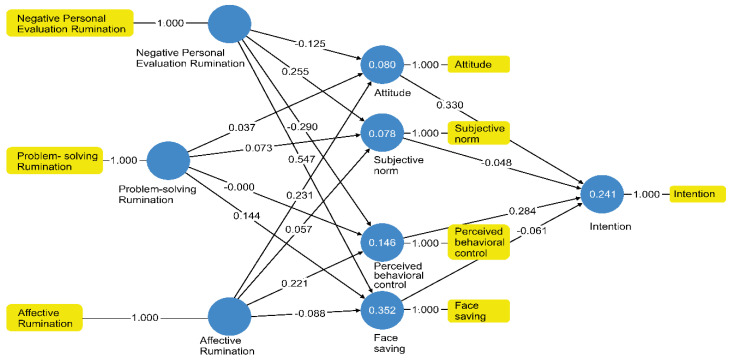
Partial Least Squares (PLS) Path Analysis for the Modified Theory of Planned Behavior and Leisure Rumination.

In this study, a model of the influencing factors that affect leisure rumination was constructed based on the modified theory of planned behavior, and the factors were empirically analyzed using the PLS structural equation method. The results of the analysis are shown in Figure 2.

Considering that the path coefficient of consumer behavior is generally recognized as the minimum value, the path coefficient of the leisure activity intention was confirmed, based on previous studies, with the leisure rumination that was regarded as a fair value [80,81,82,83]. To verify the hypotheses of this study, an analysis of the significance of the path coefficients was conducted. The analysis results obtained through the use of the bootstrapping 5000 procedure are provided in Table 5. Model suitability was shown to be SRMR: 0.046, d_ULS: 0.076, d_G: 0.022, chi-square: 40.602, and NFI: 0.910.

Figure 2 shows both the measurement model and structural model of the PLS structural equation, and the potential variables and measurement indicators are presented as circles and squares, respectively. The numbers in the circles represent the values of the coefficient of determination (R^2^), indicating the degree of variance explained by other variables.

H2, H4, H6-1, H6-2, H6-2, H7-2, and H7-4 were not supported, as they did not show significant results. Only H6-4 and H5-1 were confirmed at the 0.05% significance level, while the remaining hypotheses were supported at the 0.01% level.

The results of this study showed that the theory of planned behavior is an effective model for explaining leisure rumination. Overall, attitude toward leisure activity had the most significant effect on leisure activity intention, followed by behavioral control with a medium effect size. The indirectly measured results of the relationship between the four influencing factors and the factors of leisure rumination showed that face-saving had the most significant effect on problem-solving rumination, followed by subjective norm, attitude, and behavioral control in decreasing order. Finally, in the affective rumination factor of leisure rumination, the factors of attitude and behavioral control showed significant effects.

## 5. Discussion

This study aimed to determine the relationship between leisure rumination in Korean employees according to the modified theory of planned behavior. That is, the study was conducted to confirm the new concept of leisure rumination as a leisure activity. Therefore, this study employed the PLS research method to confirm whether leisure rumination acts as a positive factor to relieve everyday stress. Some of these hypotheses were unsupported; however, the influence of leisure rumination in the overall research model was verified through the modified theory of planned behavior.

First, for employees who engaged in leisure activities, the attitude and behavioral control factors of the modified theory of planned behavior had a positive effect on their intention. As previous studies by Kim [33], Ménard et al. [84], and Ajzen [27] have suggested, the subjective possibility of behavioral intention, when the level of one’s positive attitude toward engaging in leisure activities is high, the intention to engage in leisure activities is likewise high. In other words, leisure activity intention includes the attitude and behavioral control factors that affect behavior, and generally, the higher the attitude factor, the higher the behavior performance. Therefore, the attitudes of Korean employees toward continued engagement in leisure activity behaviors and behavioral control are expected to affect their intention to perform said leisure activities.

Second, among the factors of the modified theory of planned behavior, the problem-solving rumination factor of leisure rumination was found to have a positive effect on the face-saving factor. The results of this study showed that problem-solving rumination affects face-saving, which places importance on the psychological characteristics of Koreans and the order of rank in Korean society [28,29,30,33,44]. Moreover, problem-solving rumination involves thoughts related to present and future leisure activities, affects the intention of employees to continue leisure activities, and has a positive effect on life [45] and the acknowledgment of one’s face-saving behavior [46]. Therefore, problem-solving rumination is expected to have an influence on the intention of employees to continue participating in leisure activities, which may then reduce their work stress.

Third, the negative personal evaluation factor of leisure rumination was found to have effects on subjective norm, face-saving, attitude, and behavioral control. Kang and Kim [21] showed that participation in leisure activities is often met with positive evaluation by others, which consequently affects the continuation of that behavior. Problem-solving rumination is perceived as a factor that negatively affects the attitude and behavioral control factors of the modified theory of planned behavior and positively affects subjective norm and face-saving [85,86]. People with high problem-solving rumination could consider leisure activities as a way to relieve work stress, but have difficulty participating in such activities, because of the possible negative evaluation from others. Therefore, the negative factors of interpersonal evaluation confirmed that the evaluation of others negatively affected attitudes and perceptions of behavioral control through thoughts of leisure activities, and thinking of leisure through positive influences on face-saving and subjective norms was the result of work stress. Therefore, it was confirmed that negative personal evaluation factors affect leisure activities for employees.

Fourth, the affective rumination factor of leisure rumination was found to have a positive effect on the attitude and behavioral control factors of the modified theory of planned behavior. Affective rumination refers to employees experiencing a positive emotional state and mental recovery during work or in daily life through thoughts of leisure activity [17,21,87,88]. Moreover, this study identified that affective rumination may reduce negative emotional experiences by enabling employees to resolve work stress more actively through the pleasant recollection of leisure activities [4]. In other words, the affective rumination factor of leisure rumination seems to have a positive effect on the intention to continue leisure activities, through positive thoughts in daily life and behavioral control. Therefore, employees can sustain a positive work-life balance through affective rumination, which has a positive effect on the formation of intention to continue leisure activities and on behavioral control.

## 6. Conclusions

This study explored the meaning of leisure rumination in daily life based on the existing modified theory of planned behavior. Through this study, a relationship between the modified theory of planned behavior and leisure rumination was identified. To date, the results of studies on rumination have been used as a basis for identifying negative phenomena occurring after traumatic accidents. However, this study aimed to change the perception that ordinary leisure thoughts and activities are unnecessary, by verifying both positive and negative factors caused by leisure rumination, as confirmed by Kim [17] and Kang and Kim [21]. Through this, it could be considered a concept of relaxation by looking for a developmental direction through leisure rumination and experiencing happiness in daily life. In other words, leisure rumination is expected to affect the continuation of positive activities and leisure activities, which could have positive effects on work addiction and burnout stress. Thus, leisure activity through leisure rumination is considered an energizing element in the daily lives of employees that can relieve work stress. However, since this study was conducted on various leisure activities, additional verification of the relationships between leisure rumination on specific leisure activities and social and psychological factors is needed.

This study has several limitations. First, this study was conducted to determine the meaning of leisure rumination through the social and psychological factors of planned behavior. However, since this study was conducted on general leisure activities of the participants, the present data were insufficient to analyze the significance of leisure rumination for each leisure activity in detail. Although a positive significance of leisure rumination has been established through this study, the positive significance of each leisure activity should be reconfirmed. Second, since this study was conducted on employees, studies on diverse populations are needed. The positive aspects of leisure activities were identified, as the study was conducted on employees continuing such activities; however, it is necessary to analyze the importance of leisure rumination during schoolwork in children and adolescents who spend considerable time on studies and lack experience in leisure activities. Furthermore, leisure-related majors and the concept of studying leisure activities should be expanded and provided as basic data through analysis of the significance of leisure rumination in the daily lives of older adults.

## Figures and Tables

**Table 1:** Validity of the Leisure Rumination Scale.

Leisure Rumination Vriable	Indicators	Loadings	Cronbach’s Alpha	Composite Reliability	AVE
Problem-solving rumination	PR1	0.701	0.823	0.881	0.650
	PR2	0.791			
	PR3	0.855			
	PR4	0.868			
Negative Personal Evaluation Rumination	NP1	0.865	0.904	0.931	0.773
	NP2	0.879			
	NP3	0.861			
	NP4	0.911			
Affective Rumination	AR1	0.886	0.888	0.922	0.747
	AR2	0.861			
	AR3	0.902			
	AR4	0.803			

AVE: Average Variance Extracted, PR: Problem-Solving Rumination, NP: Negative Personal Evaluation Rumination, AR: Affective Rumination.

**Table 2:** Reliability and Validity of the Leisure Rumination Scale.

Leisure Rumination Vriable	Problem-Solving Rumination	Negative Personal Evaluation Rumination	Affective Rumination
Problem-solving Rumination	0.806		
Negative Personal Evaluation Rumination	0.128	0.879	
Affective Rumination	0.299	−0.113	0.864

**Table 3:** Validity of the Modified Theory of Planned Behavior Scale.

Modified Planned Behavior Theory Variable	Indicators	Loadings	Cronbach’s Alpha	Composite Reliability	AVE
Intention	IN1	0.952	0.969	0.980	0.941
	IN2	0.984			
	IN3	0.975			
Subjective norm	SN1	0.851	0.839	0.903	0.756
	SN2	0.870			
	SN3	0.888			
Face-saving	FS1	0.883	0.901	0.938	0.835
	FS2	0.939			
	FS3	0.919			
Attitude	AT1	0.913	0.892	0.925	0.754
	AT2	0.863			
	AT3	0.823			
	AT4	0.872			
Perceived behavioral control	PBC1	0.851	0.789	0.876	0.703
	PBC2	0.870			
	PBC3	0.888			

AVE: Average Variance Extracted, SN, Subjective Norms; PBC, Perceived Behavioral Control; AT, Attitude; INT, Intention; FS, Face-Saving.

**Table 4:** Reliability and Validity of the Modified Theory of Planned Behavior Scale Using the Fornell–Larcker Criterion.

Modified Planned Behavior Theory Variable	Intention	Subjective Norm	Face-Saving	Attitude	Perceived Behavioral Control
Intention	0.970				
Subjective norm	−0.077	0.870			
Face-saving	−0.190	0.291	0.914		
Attitude	0.396	−0.043	−0.180	0.868	
Perceived behavioral control	0.354	−0.009	−0.212	0.173	0.839

**Table 5:** Exploratory Factor Analysis of the Modified Theory of Planned Behavior.

Hypothesis	Path	Path Coefficient	*t*-Value	Result
Hypothesis 1	Attitude→Intention	0.330	6.776 **	Supported
Hypothesis 2	Subjective norm→Intention	−0.048	0.984	Rejected
Hypothesis 3	Perceived Behavioral control→Intention	0.284	4.731 **	Supported
Hypothesis 4	Face-saving→Intention	−0.061	1.274	Rejected
Hypothesis 5-1	Negative Personal Evaluation Rumination→Attitude	−0.125	2.281 *	Supported
Hypothesis 5-2	Negative Personal Evaluation Rumination→Subjective norm	0.255	4.810 **	Supported
Hypothesis 5-3	Negative Personal Evaluation Rumination→Perceived Behavioral control	−0.290	5.829 **	Supported
Hypothesis 5-4	Negative Personal Evaluation Rumination→face-saving	0.547	10.768 **	Supported
Hypothesis 6-1	Problem-Solving Rumination→Attitude	0.037	0.609	Rejected
Hypothesis 6-2	Problem-Solving Rumination→Subjective norm	0.073	1.297	Rejected
Hypothesis 6-3	Problem-Solving Rumination→Perceived Behavioral control	−0.000	0.005	Rejected
Hypothesis 6-4	Problem-Solving Rumination→Face-saving	0.144	2.961 *	Supported
Hypothesis 7-1	Affective Rumination→Attitude	0.231	4.090 **	Supported
Hypothesis 7-2	Affective Rumination→Subjective norm	0.057	1.046	Rejected
Hypothesis 7-3	Affective Rumination→Perceived Behavioral control	0.221	4.090 **	Supported
Hypothesis 7-4	Affective Rumination→Face-saving	−0.088	1.448	Rejected

* *p* < 0.05, ** *p* < 0.01.

## Data Availability

The data presented in this study are available on request from the corresponding author.
